# Influence of maternal diet on offspring survivorship, growth, and reproduction in a sheetweb spider

**DOI:** 10.1242/bio.056846

**Published:** 2020-11-06

**Authors:** Lelei Wen, Zengtao Zhang, Shichang Zhang, Fengxiang Liu, Xiaoguo Jiao, Daiqin Li

**Affiliations:** 1 State Key Laboratory of Biocatalysis and Enzyme Engineering, Centre for Behavioural Ecology and Evolution (CBEE), School of Life Sciences, Hubei University, Wuhan, 430062, Hubei, China; 2 Department of Biological Sciences, National University of Singapore, 14 Science Drive 4, Singapore 117543

**Keywords:** Fitness, *Hylyphantes graminicola*, Maternal diet, Reproduction, Spider, Survival

## Abstract

Prey vary dramatically in quality, and maternal diet is generally assumed to substantially influence offspring survivorship, growth, and reproduction in spiders. Numerous studies that have tested this hypothesis have focused exclusively on parental generation or have considered relatively few fitness components of juvenile offspring. However, maternal diet may have a substantial effect on fitness performance beyond juvenile offspring. Here, we investigated the influence of one-time maternal feeding on multiple offspring fitness components, including the survival rate and growth of juvenile offspring as well as the mating and reproductive success of adult offspring in *Hylyphantes graminicola*, a sheetweb spider with an extremely short lifespan (∼1 month). We fed field-collected adult female spiders two different diets only once immediately before oviposition: midges (*Tendipes* sp.) only (MO) or flies (*Drosophila melanogaster*) only (FO). Juvenile offspring of MO females had significantly higher survival rate, faster growth, and larger male size at maturity than FO offspring. Although maternal diet did not significantly influence mating behavior or fecundity of female offspring overall, those of MO females laid eggs earlier and their eggs also hatched earlier and had a higher hatching rate than those of FO females. Intriguingly, one-time maternal feeding was sufficient to have such an influence on offspring fitness even beyond juvenile offspring in *H*. *graminicola*. This one-time maternal effect may be widespread in other spiders and other invertebrates with a short lifespan.

This article has an associated First Person interview with the first author of the paper.

## Abstract (precis)

**Summary:** A single meal immediately before oviposition is sufficient to influence offspring survival, growth, and daughters' reproduction in the sheetweb spider *Hylyphantes graminicola*.

## INTRODUCTION

Spiders are one of the most diverse and abundant generalist predators (Wilder, 2011). The often limited prey (in both quantity and quality) in nature has a crucial impact on spider survival, growth, and reproduction (Wise, 2006; Wilder, 2011; Toft, 2013). Previous studies that have mainly focused on the effects of a particular prey species on spider fitness performance revealed that spider fitness varied substantially depending on the prey species (Li and Jackson, 1996a,b, 1997; Bilde and Toft, 2000; Sigsgaard et al., 2001; Rickers et al., 2006; Wilder, 2011; Toft, 2013; Líznarová and Pekár, 2016; Salomon et al., 2011; Wilder, 2011; Toft, 2013; Johnson et al., 2014). For example, linyphiid spiders (*Erigone atra*) had a higher survivorship and growth rate when raised on *Isotoma anglicana* collembolas than those raised on *Folsomia fimetaria* collembolas (Marcussen et al., 1999). In addition, wolf spiders (*Schizocosa*) grew slowly and even died when raised on *Folsomia* *candida* collembolas, but had a high survival rate and grew more rapidly when raised on *Tomocerus bidentatus* collembolas (Toft and Wise, 1999). These studies suggest that different prey species may differ in terms of the nutrients that are utilizable and beneficial for any given spider species, thus showing differential influences on spider fitness (Wilder, 2011; Toft, 2013).

At the same time, however, any given species of prey may vary in quality for a single spider species, depending on the developmental stage of a spider, because different stages may require different critical nutrients (Toft, 1995, 2005, 2013). For example, fruit flies (*Drosophila melanogaster*) are the most common prey used to raise spiders in laboratories and to evaluate the influence of prey quality (e.g. macronutrients) on spider fitness performance, and previous studies have demonstrated that fly quality varies dramatically by life stage of many spider species (Toft and Wise, 1999; Oelbermann and Scheu, 2002; Peng et al., 2013). For instance, the flies tend to be suitable for the growth of early instars but not later stages: over time, spiders grow more slowly and may even cease growth and die during molting (Lowrie, 1987; Toft and Wise, 1999; Higgins and Rankin, 2001; Mayntz and Toft, 2001; Oelbermann and Scheu, 2002). This suggests that spiders may require different species of prey or a mixture of different species to obtain critical nutrients or attain a balance of nutrients during the different developmental stages (Greenstone, 1979; Mayntz and Toft, 2001). For this reason, previous studies have primarily focused on spider survivorship and growth of the current generation.

The prey on which mother spiders feed (i.e. maternal diet) have substantial effects on the fitness of juvenile offspring (Wilder and Rypstra, 2008; Salomon et al., 2011; Wilder, 2011; Toft, 2013; Johnson et al., 2014; Wilder and Schneider, 2017). For example, in an orb-web spider (*Argiope bruennichi*), juvenile offspring of females whose diet was supplemented with essential amino acids survived longer than those of other treatments (i.e. water control, dietary essential fatty acids, nonessential amino and fatty acids) (Wilder and Schneider, 2017). Rickers and colleagues (2006) investigated the reproduction of female wolf spiders (*Pardosa lugubris*) that had fed on prey of different qualities using stable isotope analyses. Their results suggested that dietary nutrients can be routed almost exclusively to eggs. Interestingly, toxic collembolas (*F*. *candida*) even prevented female spiders from absorbing and assimilating the nutrients from nontoxic prey (Rickers et al., 2006). In a linyphiid spider (*Erigone atra*), the body size of hatchlings varied dramatically with maternal diet (Toft, 1995). In another linyphiid spider, *Dicymbium brevisetosum*, survivorship of offspring of females raised on normal fruit flies (reared on plain medium) was lower than that of offspring of females that were raised on enriched fruit flies (reared on nutritionally improved medium) (Bilde and Toft, 2000). These studies highlight that the influence of prey on spider fitness cannot be fully understood without taking the effects of maternal diet on offspring fitness into account (Bilde and Toft, 2000; Salomon et al., 2011; Johnson et al., 2014; Wilder and Schneider, 2017). However, previous studies have only investigated the effect of maternal diet on one or a few offspring fitness components and have rarely explored beyond juvenile stages of offspring.

In this study, we investigated the effects of maternal diet on multiple fitness-related consequences of offspring including the impacts on adult offspring. We used a sheetweb spider, *Hylyphantes graminicola*, as a model system, and considered survivorship and growth of juvenile offspring as well as mating success and reproduction of adult offspring. *Hylyphantes graminicola* (Araneae: Linyphiidae) is distributed widely in Asia and is one of the most important natural enemies of insect pests in agricultural ecosystems (Zhao, 1993; Peng et al., 2013). It is a generalist predator with a very small body size (body length: 2–4 mm) and an extremely short life cycle (from egg-laying to sexual maturation: ∼1 month under an optimal range of temperatures) (Zhao, 1993; Li and Jackson, 1996a,b). Females usually undergo five instars to reach sexual maturity and the last instar takes about less than 1 week. Mated females usually lay their eggs in a week (Zhao, 1993). Previous observations have suggested that *H*. *graminicola* juvenile offspring have an extremely high survivorship when their mothers are fed midges (*Tendipes*) (unpublished data). Because *H*. *graminicola* has a very short life cycle and adult females can lay eggs shortly after mating (in 5 days; Zhao, 1993), we hypothesized that one-time feeding by adult females immediately before oviposition with high-quality prey would be sufficient to influence offspring fitness performance. We tested this hypothesis by feeding field-collected adult females with midges (*Tendipes* sp.) only (MO) or fruit flies (*D*. *melanogaster*) only (FO), and only once.

## RESULTS

### Juvenile offspring survival rate and developmental duration

**Fig. 1.**
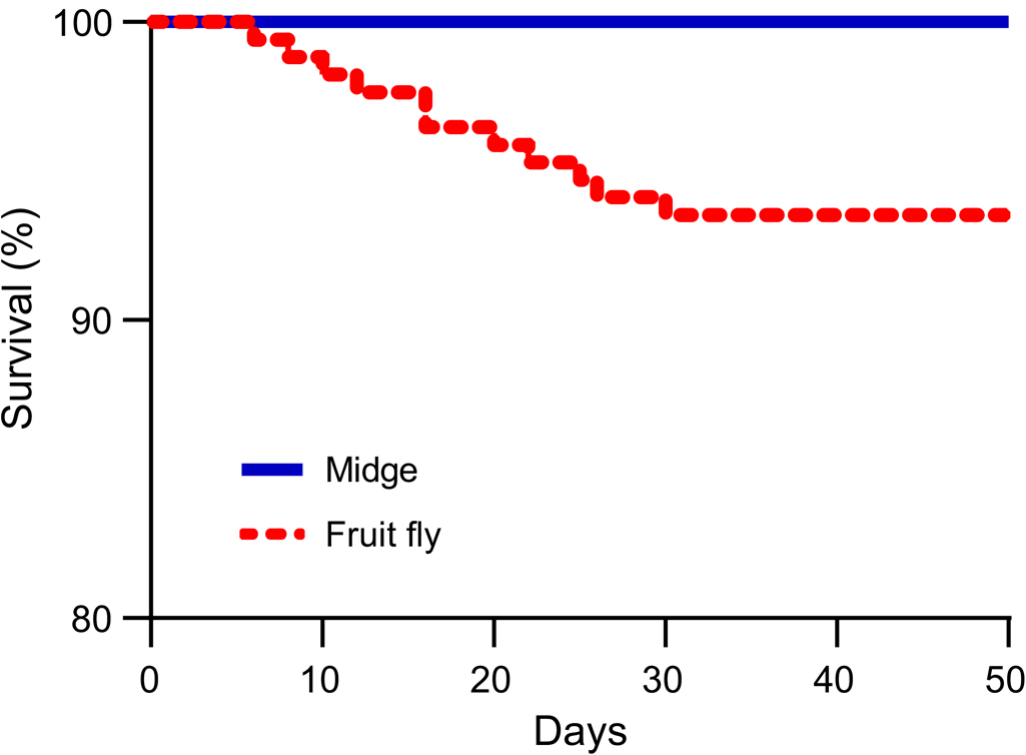
**The survivoship (%) of *H. graminicola* juvenile offspring reared on the two maternal diets.** Kaplan–Meier survival curve of the independent effect of a maternal diet comprising fruit flies only (*D*. *melanogaster*, *N*=126) or midges only (*Tendipes* sp., *N*=170) on the survival rate of *H.* *graminicola* juvenile offspring (log-rank test: χ^2^=8.40, d.f.=1, *P*=0.004).

The percentage of first-instar juvenile offspring that survived to maturity differed between the two maternal diet groups (Fig. 1), being significantly higher for MO offspring (100%) than for FO offspring (94%) (log-rank *χ*^2^=8.40, d.f.=1, *P*=0.004).

**Fig. 2.**
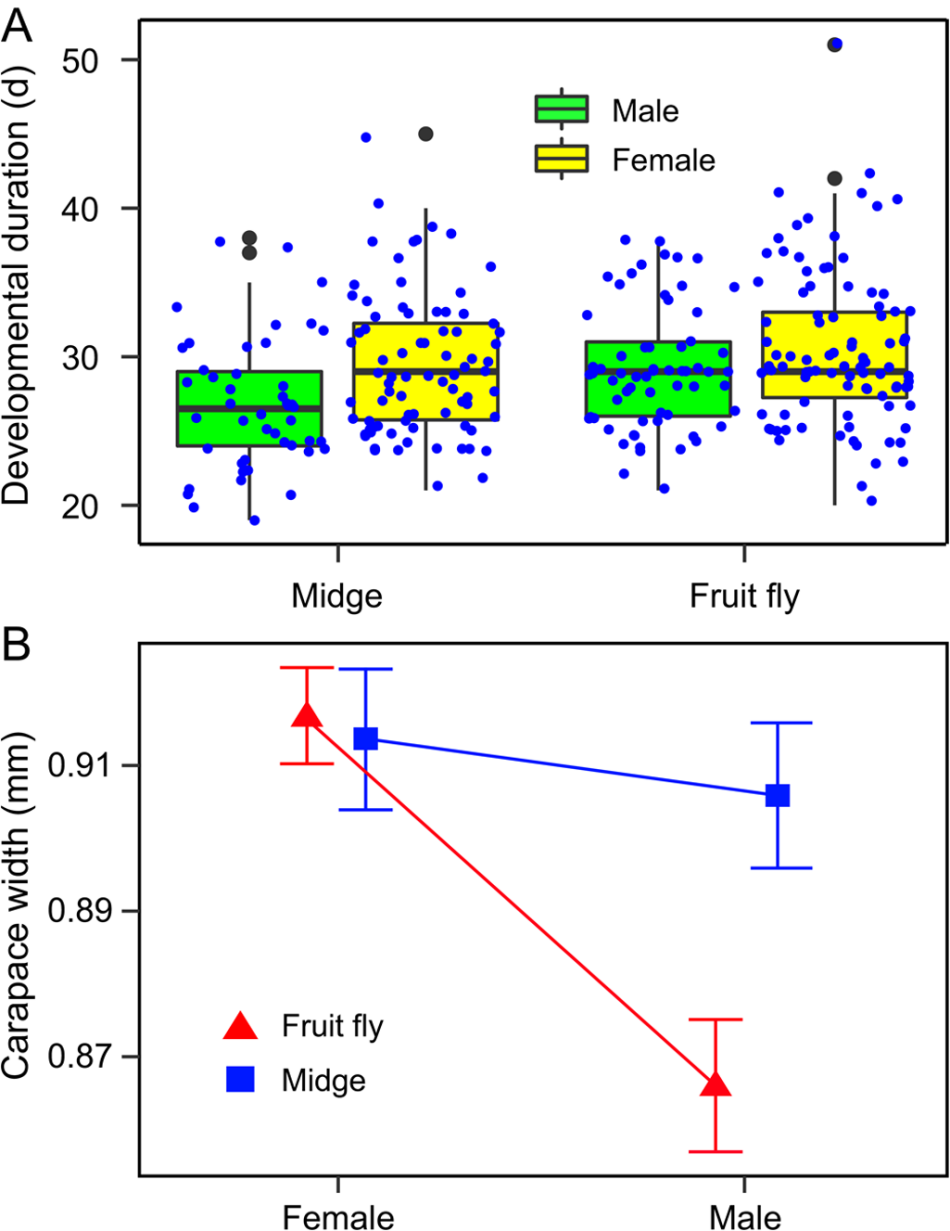
**(A) Boxplots of the developmental duration (d) (midge only: *N*=126; fly only: *N*=159) and (B) mean (±s.e.) carapace width (mm) (midge only: *N*=50; fly only: *N*=127) of *H.graminicola* offspring juveniles.** Female spiders were fed once on midges (*Tendipes* sp.) or fruit flies (*D.* *melanogaster*) immediately before oviposition. Offspring juveniles were fed fruit flies.

Mother identity as a random effect had no significant effects on juvenile offspring developmental time as the model without the random effects fitted better than the model with the random effects (*χ*^2^=9.31, d.f.=1, *P*=0.002) (Table 1). Overall, male offspring reached sexual maturity significantly earlier than females (*β*=−0.06, *t*=−3.50, *P*=0.005; Fig. 2A). In group comparisons, MO offspring reached maturity significantly earlier than the other group (*β*=0.05, *t*=−2.06, *P*=0.049; Fig. 2A). However, there was no significant interaction between maternal diet and sex (*χ*^2^=2.46, d.f.=1, *P*=0.117) (Fig. 2A) (Table 1).

**Table 1.**
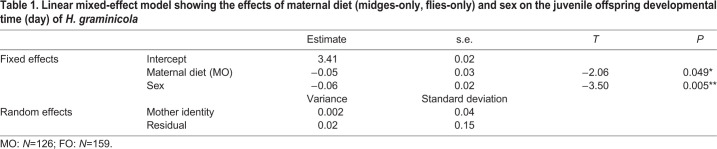
**Linear mixed-effect model showing the effects of maternal diet (midges-only, flies-only) and sex on the juvenile offspring developmental time (day) of *H. graminicola***

Similarly, mother identity had no significant effects on the body size of offspring at sexual maturity as the model without the random effects was better than the model with the random effects (*χ*^2^=12.79, d.f.=1, *P*<0.001) (Table 2). Sex (*β*=−0.051, *t*=−5.13, *P*<0.001) and the interaction between maternal diet and sex (*β*=0.05, *t*=2.55, *P*=0.012), but not maternal diet (*β*=−0.01, *t*=−0.34, *P*=0.734), had significant effects on the body size of offspring at sexual maturity (Fig. 2B) (Table 2). Female offspring were significantly larger than males in the FO group (*P*<0.001), but there was no significant difference in body size between the sexes within the MO group (*P*=0.632). There was no significant difference in female offspring body size at maturity between the two maternal diet groups (*P=*0.844), but there was significant difference in male offspring body size at maturity between the two maternal diet groups (*P=*0.003).

**Table 2.**
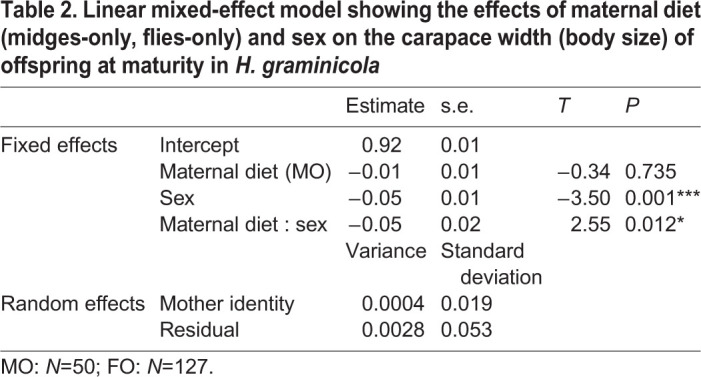
**Linear mixed-effect model showing the effects of maternal diet (midges-only, flies-only) and sex on the carapace width (body size) of offspring at maturity in *H. graminicola***

### Mating and reproduction of adult offspring

**Fig. 3.**
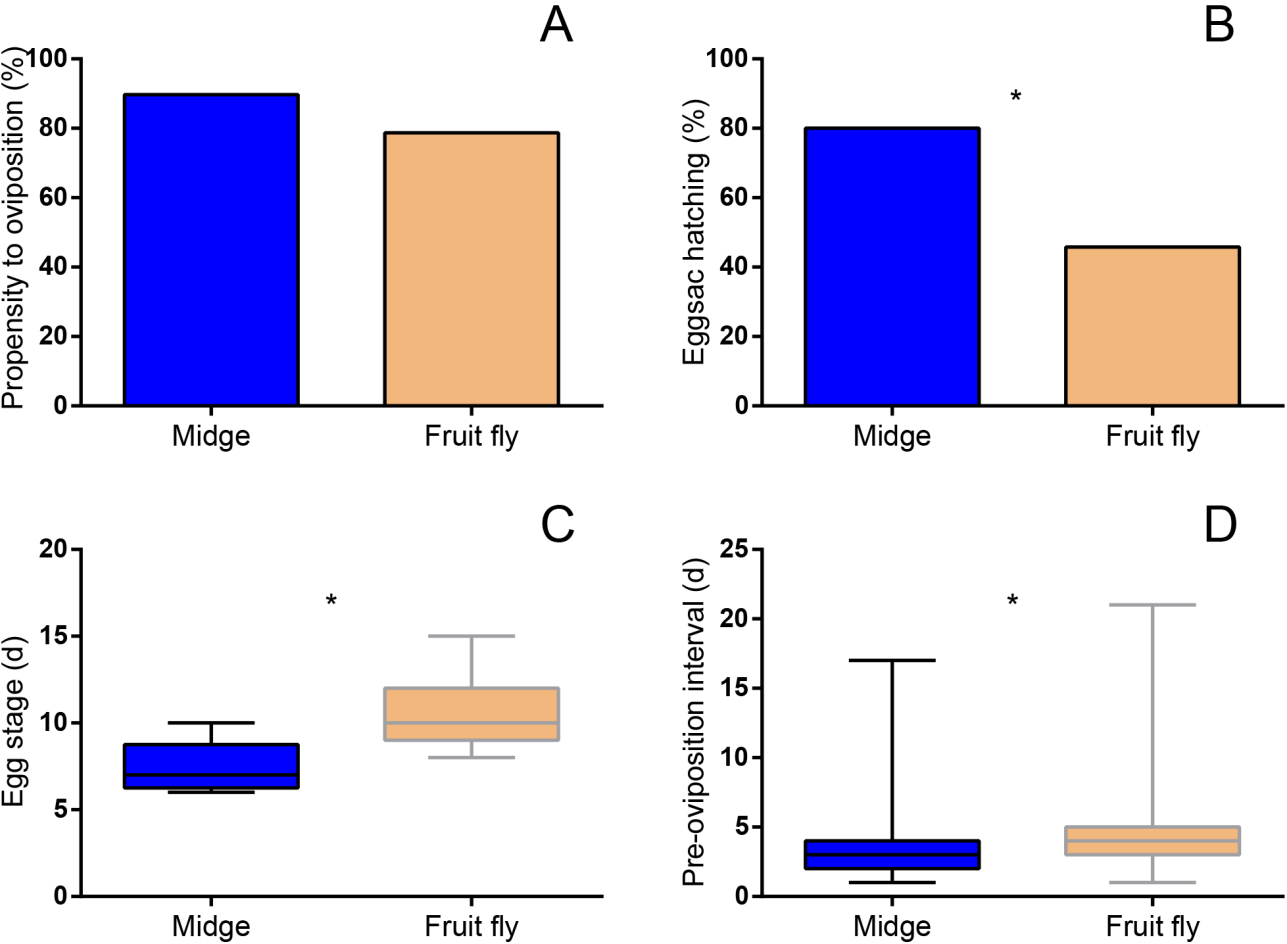
**Comparison of reproductive parameters of female offspring *H.graminicola* between the two maternal diet groups (MO; FO).** (A) Propensity to oviposit (MO: *N*=39; FO: *N*=75); (B) percentage of hatched first egg sacs (number of first egg sacs that hatched/total number of egg sacs produced; MO: *N*=35; FO, *N*=59); (C) egg stage in days (MO: *N*=28; FO: *N*=27); (D) pre-oviposition interval in days (MO: *N*=35; FO: *N*=59). Asterisks indicate significant differences.

Maternal diet did not have a significant effect on mating success (chi-square test for independence: *χ*^2^=0.009, d.f. =1, *P*=0.925), mating latency (Wilcoxon rank sum test: *W=*1686, *P*=0.174), copulation duration (*W*=1499, *P*=0.832), or the number of mating bouts (*W* =1423, *P*=0.785) (Table 3). There were no significant differences in propensity to oviposit between the two maternal diet groups (*χ*^2^=2.18, d.f.=1, *P*=0.140; Fig. 3A). However, female offspring from the MO group laid eggs significantly earlier (*W*=1341, *P*=0.014; Fig. 3D) and their eggs also hatched significantly earlier (*W*=704, *P*<0.001; Fig. 3C) than those from the FO group. Meanwhile, more female offspring from the MO group produced viable egg sacs than those from the FO group (*χ*^2^=10.61, d.f.=1, *P*=0.001; Fig. 3B). Although the fecundity of female offspring was not significantly different between the two diet groups (*W*=809, *P*=0.081; Table 4), more eggs produced by female offspring from the MO group hatched (*W*=365, *P*<0.001; Table 4) with a higher hatching rate (*W*=361, *P*<0.001; Table 4) than those produced by female offspring from the FO group.

**Table. 3.**
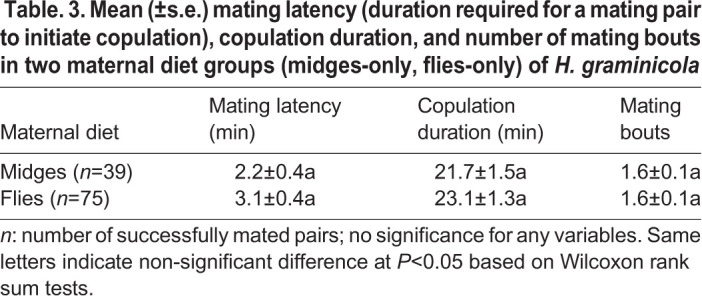
**Mean (±s.e.) mating latency (duration required for a mating pair to initiate copulation), copulation duration, and number of mating bouts in two maternal diet groups (midges-only, flies-only) of** ***H. graminicola***

**Table. 4.**
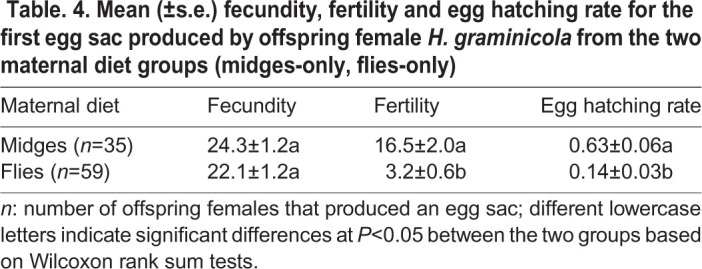
**Mean (±s.e.) fecundity, fertility and egg hatching rate for the first egg sac produced by offspring female *H. graminicola* from the two maternal diet groups (midges-only, flies-only)**

## DISCUSSION

Our study supports the hypothesis that maternal diet has a strong influence on juvenile offspring survival, development rate, and size at maturity, as well as adult female offspring's reproductive output in the sheetweb spider *H*. *graminicola*. Intriguingly, our study demonstrates that one-time feeding of field-collected adult female spiders immediately before oviposition is sufficient to influence offspring fitness across two generations. Even more, our results suggest that genetic background and offspring diet may not be the main factors influencing offspring survival, growth, and reproduction, as all spiders were collected from the same population and were randomly divided into the two maternal diet groups. In addition, our data analyses showed that mother identity included as a random effect in the models had no significant effects on offspring growth rate and the size at maturity. Furthermore, all offspring were raised on the same type and amount of fruit flies. Thus, the detected differences in offspring fitness components between the two groups resulted from the different maternal diets.

Spiders feed using extraoral digestion (Cohen, 1995), which can effectively separate edible nutrients from inedible parts of prey and allow spiders to maximize their nutrient intake (Wilder, 2011). Field-collected midges seem to be much more nutrient rich for *H*. *graminicola* than laboratory-cultured fruit flies. Laboratory-raised fruit flies usually have very high levels of lipids in the abdomen and a considerable high-protein but inedible exoskeleton, whereas midges are rich in essential amino acids, which are crucial for molting during the later life stages of spiders (Greenstone, 1979). Female *H*. *graminicola* may thus acquire much more protein with a better profile of essential amino acids from field-collected midges than laboratory-cultured fruit flies.

With a very short life cycle and a very short pre-oviposition interval (Zhao, 1993), adult *H*. *graminicola* females may invest greatly in their offspring via eggs. There is growing evidence that female spiders can route dietary nutrients almost exclusively to egg production (Rickers et al., 2006; Blamires, 2011). Blamires (2011) found that female spiders fed high-protein but low-energy flies produced eggs that had higher energy content than females fed low-protein but high-energy flies. Essential amino acids may thus provide crucial nutrients involved in allocating nutrients to eggs (Wilder and Schneider, 2017). Hence, in our study, field-collected adult *H*. *graminicola* females that acquired well-balanced nutrients may have obtained a much higher level of proteins and essential amino acids from midges, and then routed them to their eggs (the effects of which extended beyond eggs), although they fed only once before oviposition.

Spiders often die before maturing when raised on a diet composed of only one species (Riechert and Harp, 1987; Uetz et al., 1992). However, this did not happen in the present study. Approximately 94–100% of juvenile offspring from both groups survived to maturity. We found that MO offspring had significantly higher survival rates than FO offspring. This suggests that a single quality feeding immediately before oviposition can transfer enough critical nutrients to the next generation. Several studies that have investigated the maternal effects on the survivorship of juvenile offspring have reported similar results (Salomon et al., 2011; Wilder and Schneider, 2017; Chen et al., 2018). These studies indicate that proteins and essential amino acids in consumed prey are positively related with spider survival. Thus, midges collected from the wild may provide certain critical nutrients that are absent in lab-cultured fruit flies, and thus support high survivorship in *H*. *graminicola*. However, our study is different from previous studies in that previous studies have only examined the effects of maternal factors on offspring survival for only one or a few instars of development. We also considered the effects on adults.

We found that MO offspring developed faster and attained larger size (in males) at maturity than FO offspring. Mayntz and Toft (2001) found that juvenile spiders grew much faster when fed a basic medium diet supplemented with 19 different amino acids, fatty acids, cholesterol, and commercial dog food. In Jensen et al. (2011), prey containing high levels of protein shortened juvenile development time and increased body size in the wolf spider *Pardosa amentata* although the juveniles were observed for only a few instars. In the jumping spider *Phidippus audax*, however, Wiggins and Wilder (2018) found that high-lipid prey was associated with larger body size (tibia/patella length and posterior-lateral eye width) and heavier weight. In our experiments, the positive effects of mother diet on juvenile growth were mediated by some substances in midges after a single feeding. It is generally accepted that prey protein, rather than other nutrients, promotes spider growth (Blamires et al., 2009; Jensen et al., 2011; Wilder, 2011). Perhaps the midges provide much higher levels of essential amino acids or proteins for *H*. *graminicola* than fruit flies. It should be noted that, in our study, only male offspring body size was markedly affected by maternal diet. This may be because male spiders usually eat less than females, and during our experiments all offspring were given equal amounts of food of the same quality.

Our study shows that MO daughters had significantly better reproductive success than FO daughters, with shorter pre-oviposition and latency to egg hatching and the production of more fertile and viable eggs. One possibility is that the midges provide offspring more proteins that promote their reproduction (Blamires, 2011; Bressendorff and Toft, 2011; Salomon et al., 2011). For example, Wilder and Rypstra (2008) found that, although mating success and egg production of *Pardosa milvina* females are not significantly affected by diet quality, females produce egg sacs more quickly when reared on a high-quality (high nitrogen) diet. Rickers et al. (2006) used isotope tracer experiments to confirm the effective accumulation of nitrogen in egg sacs. Taken together, these results suggest that prey with high levels of protein may accelerate egg production. We expect that field-collected midges may provide more proteins than laboratory-cultured fruit flies. The further analysis of nutritional composition will thus be able to confirm this.

We observed that MO daughters had higher fertility and egg hatching rates than FO daughters. Similar results were reported in the seven-spotted lady beetle *Coccinella septempunctata* in a study by Ugine et al. (2019), who concluded that cholesterol is indispensable for male lady beetles to produce sufficient viable sperm to inseminate females. The same may be true for male *H*. *graminicola*. Spiders are unable to synthesize cholesterol *de novo* and only acquire it from their prey (Jing and Behmer, 2020). The fruit flies in our experiment were cultured in a medium composed mainly of corn meal, sucrose, and yeast extract powder, which is most likely deficient in cholesterol. Hence, overall, we can ascribe all observed differences in reproduction parameters to the effects of maternal diet on male offspring. Male size seems to be positively correlated with the fertility of the females with which they mate, although this has not been thoroughly tested.

In conclusion, the consumption of a single high-quality meal by a mother immediately before oviposition can have a lasting positive effect on the survival, growth and reproduction of offspring in *H*. *graminicola*. More specifically to our study, these positive transgenerational effects suggest that supplementary feeding of midges is beneficial for mass breeding of *H*. *graminicola* spiders.

## MATERIALS AND METHODS

### Collection and manipulation of maternal diet

We collected *H*. *graminicola* as adult females in June 2016 from a single corn field in Longmen Town (34°34′ N; 112°29′ E), Luoyang City, Henan Province, China. We did this because the mortality of laboratory-raised spiders after a few generations is usually high, whereas field-collected adult females from the same habitat would have similar genetic background and balanced nutrients, and thus should produce viable offspring. We brought them back to the laboratory and kept them individually in glass tubes (diameter×length: 20×60 mm). We housed all the spiders in an incubator with controlled environmental conditions (temperature: 25±0.5°C; relative humidity: 60–80%; light regime: 14 h:10 h). We provided them with water *ad libitum* using a piece of water-dampened sponge placed at the bottom of the glass tubes.

To test the effects of one-time maternal diet on offspring fitness performance, we randomly assigned the collected spiders into two diet groups: FO and MO. On the second day of their collection, FO females (*n*=50) were fed 15 mg (wet mass) fruit flies only and MO flies (*n*=40) were fed 15 mg (wet mass) adult midges only. Each group was fed only one time immediately before oviposition as adult females are known to lay eggs in 5 days after mating (Zhao, 1993). Fruit flies were raised on corn medium and adult midges were caught locally. Most females laid their first egg sac within a week after their feeding, and their eggs hatched within a week. The randomly selected first egg sacs produced by the females from each diet group (MO: *n*=13; FO: *n*=8) provided the hatchlings for subsequent experiments.

### Offspring survivorship and developmental time

To determine the influence of the one-time maternal feeding on offspring survival rate, developmental duration, and body size at sexual maturity, following hatching and dispersal, we isolated first instar spiderlings (for definition see Hallas, 1988), kept them individually in glass tubes (20×60 mm) and divided them by maternal feeding group (MO, *n*=126; FO, *n*=170). All spiderlings were fed fruit flies every 4 days except that the first instar spiderlings were fed frozen fruit flies because they had difficulty catching live flies. The rearing conditions for all spiderlings were identical (temperature: 25±0.5°C; relative humidity: 60–80%; light regime: 14 h:10 h).

We monitored spiderling survival daily until sexual maturity. Because *H*. *graminicola* has a very short life cycle, we did not calculate the survival rate or developmental duration for each instar. We calculated the offspring survival rate for the total juvenile period between the first instar and sexual maturity for each maternal diet group. Then survival was calculated as a percentage of the total number of individuals that survived to maturation. The total juvenile developmental duration (the time elapsed between the first instar and maturation) was recorded for each offspring individual in each group. We measured the carapace width (CW) at maturity for each individual under a microscope (Leica M205 C; Leica Microsystems GmbH, Wetzlar, Germany) to the nearest 0.01 mm and used it as an indicator of size. Then offspring spiders from each group that reached sexual maturity were used in subsequent mating and reproduction experiments.

### Mating behavior and reproduction of adult offspring

To examine the effects of maternal diet on adult offspring mating behavior, we paired randomly selected females and males within the same group (MO: *n*=50; FO: *n*=97) when the post-maturation age of female offspring was 7–10 days. We randomly selected a non-sibling male and introduced it gently into a female's rearing glass tube. If the pair did not mate within 15 min, we considered the pairing a failure and gently removed the male to its original rearing tube. If the mating of the pair occurred within 15 min, we recorded mating latency (the time elapsed between the start of the mating trial and the copulation), copulation duration, and number of mating bouts. During the mating trials, several pairs exhibited a repeating pattern with short separation and re-engagement, indicating multiple mating bouts occurring in a single trial. If the pair separated for more than 5 min, we defined it as the end of the mating trial. If the pair separated for between 20 s and 5 min before the re-engagement, we recorded it as a new mating bout. In two cases, the females cannibalized the males before mating, the mating duration was less than 5 min, and the male did not appear to insert his palps into the female epigynum. We considered these cases as mating failure. Although *H*. *graminicola* is a polygamous species (Zhao, 1993), we used each spider only once in mating trials.

After mating, mated females were individually transferred to a new glass tube and were fed four living fruit flies and maintained as described above. To examine the effects of maternal diet on the reproduction of these adult females, we recorded propensity to oviposit (the percentage of adult female offspring that oviposited), pre-oviposition interval (the time interval between mating and first egg sac production), egg stage (interval between the production of first egg sac and hatching of eggs), fecundity (total number of eggs within the first egg sac), fertility (number of hatched eggs within the first egg sac), and egg hatching rate (percentage of the number of hatched eggs versus total number of eggs) of the first egg sac produced by adult female offspring in each maternal diet group.

### Data analyses

We performed all statistical analyses using R version 4.0.0 (R Core Team, 2020). We checked for normality of data using the Shapiro-Wilk test before analyses. When necessary, we transformed the data to meet the assumption of a normal distribution. We used linear mixed-effects models from the *lme4* package (Bates et al., 2015) to test the effects of maternal diet and spider sex on the developmental duration and carapace width of female and male offspring at maturation. We used *afex* package (Singmann et al., 2016) to obtain the *P*-values. We coded maternal diet treatment and offspring sex as fixed effects, and included the mother identity as a random effect. We then used likelihood ratio tests to investigate the significant differences in the developmental duration and carapace width by comparing the model with a random effect with the model without a random effect. If a significant effect was detected, then we performed *post hoc* paired comparisons with Bonferroni correction. We compared the survival rates of juvenile offspring between the two groups using the Kaplan–Meier survival analysis and log rank test. We used chi-square tests for independence to compare mating success (percentage of successfully mated pairs) and propensity to oviposit, and egg sac hatching (egg sac hatched/all egg sacs produced) between the two maternal diet groups. We also used Wilcoxon rank sum tests to determine the effects of maternal diet on mating latency, copulation duration, mating bouts, pre-oviposition interval, egg stage, fecundity, fertility, and egg hatching rate of adult offspring. All the values are reported as means±s.e. unless otherwise stated. All the reported *P*-values are two-tailed at an α level of 0.05. All essential data are available in supplementary material Table S1.

## Supplementary Material

Supplementary information
