# Supplementary material for: Influence of maternal diet on offspring survivorship, growth, and reproduction in a sheetweb spider
Source: Biol Open. 2020 Nov 6;9(11):bio056846. doi: 10.1242/bio.056846 (PMC7657467; doi:10.1242/bio.056846)
Supplement: Supplementary information [file biolopen-9-056846-s1.pdf]

Table S1

Click here to Download Table S1

Biology Open • Supplementary information
